# On the Iranian English as Foreign Language Novice and Experienced Teachers’ Attributional Styles and Professional Identity

**DOI:** 10.3389/fpsyg.2021.823815

**Published:** 2022-01-27

**Authors:** Seyed Farzad Kalali Sani, Khalil Motallebzadeh, Hossein Khodabakhshzadeh, Mitra Zeraatpisheh

**Affiliations:** ^1^Department of English, Islamic Azad University, Torbat-e Heydarieh, Iran; ^2^Department of English, Tabaran Institute of Higher Education, Mashhad, Iran

**Keywords:** professional identity, attributional styles, internal/external, permanent/transient, global/specific, teaching experience

## Abstract

Teacher professional identity (TPI) is a characteristic of a teacher, which should be developed in a long, consistent, and progressive process and usually shapes in any specific educational and social context. In addition to several factors influencing TPI, such as university education and empowerment courses, experience seems to play a significant role. Moreover, the role of psychological factors is highly undeniable in the formation and development of TPI. Attributional style (AS) is defined as the consistent way by which people can explain the reasons for the occurrence of good or bad events. Besides considering ASs as one of the crucial variables for academic success, it can be regarded as one of the aspects of shaping one’s identity in general and the teacher’s identity in particular. In order to study the relationship between AS and TPI of teachers regarding their experience, two questionnaires were distributed among 317 Iranian English as foreign language (EFL) teachers, and about 80% returned them. The researchers ran correlational analyses and they came up with a positive and significant relationship (*p* = 0.04) between TPI and teacher attributional styles (TASs) of teachers. Moreover, experienced teachers proved to have a significant difference from novice teachers regarding their TPIs. The findings of this study would hopefully be advantageous for teacher educators to educate pre-service teachers by ways of constructing identity, and they may be applicable for Iranian EFL teachers to know and focus on different aspects of their professional identity. Moreover, they learn how to manage the intervening factors and shape and empower different domains of their PI, such as teaching experience, by means of exploring and knowing their ASs.

## Introduction

Among the multiple roles any teacher is required to play in the classroom is an individual with the high level of proficiency, strong and updated tools, and information necessary to empower learners in different levels (Archana and Rani, 2017). One of the most salient aspects of considering a teacher successful is teacher identity. Among the multitude factors germane to teacher characteristics, it seems that psychological factors play the most significant role, such as attributional styles (ASs), motivation, and self-efficacy.

Professional identity can be considered as an important dimension of teachers’ characteristic, which has to be developed in a long, continuous, and ongoing process, and usually happens in contextual settings. The connection between professional identity and some personality and contextual factors has been found by Bressler and Rotter (2017). Moreover, Miller (2009) found the mutual relationship between keywords concerning identity as the main concept. Miller identified relational, transforming, negotiated, transitional, and enacted, which is mainly engaged with the ongoing and dynamic nature of the term identity. Varghese (2006) describes identity as “the influences on teachers, how individuals see themselves, and how they enact their profession in their settings.” Lace (as cited in Ivanova and Skara-Mincane, 2016, p. 2) found the predictability power of some components—awareness of one’s personality as a representative of a certain profession. Another critical component is attempting to find meaning within the respective profession, which ends up in the third component, which is the professional ambitions and motivation one has.

Among the factors, which highly influence forming beliefs, professional images, knowledge, and skills, previous and current experiences are of high priorities. All of these factors can be developed, challenged, and changed during university studies and respective field practice. Van Huizen et al. (2005, p. 8) assert “from the Vygotskian perspective, the overall aim of a teacher education program is best conceived as the development of professional identity.” Thus, it should be considered as a highly significant component in pre-service teacher education, which should provide meaningful contextualized support through guided reflection to facilitate this development (Lerseth, 2013). Ivanova and Skara-Mincane (2016) claim that teachers who develop a strong and positive professional identity are more effective, influential, and self-directed to acquire knowledge and skills, which are essential for teaching throughout their life. Lifelong learning has become a necessity to be an effective teacher.

As one of the psychological factors affecting teacher identity, it is possible to mention teacher attributional style (TAS) (Larsen and Allen, 2016). Seligman and Schulman (1986) and later Strickland (1989) defined AS as the ways by which a person explicates the permanent reasons why good or bad events usually happen. AS is a way of expressing the underlying causes of any events (either good or bad) that have roots in (a) internal or external forces, (b) being stable or transient over time, and (c) specific to the existing situation or present in other circumstances. Besides considering ASs as one of the crucial variables for academic success, it can be regarded as one of the aspects of shaping one’s identity in general and the teacher’s identity, in particular.

Regarding the role of gender, Wolleat et al. (1980) found that the attributions made by men for success in mathematics corresponded to ability, whereas women attributed their successes more to effort. Peterson and Barrett (1987, as cited in Higgins and LaPointe, 2012) found that first-year college students who made internal, stable, and global attributions for failure are at risk for poor grades. Finally, in 1988 (Kloosterman, 1988), Kloosterman tested his 1984 model and found that students made relatively more attributions in reaction to failure as opposed to success.

Dweck and Wortman (1982) mention that learners who tend to describe academic failure in terms of stable and global causes, e.g., stupid, and explain success in terms of unstable and specific causes, e.g., luck, correlate with reduced persistence, reduced beginning of tasks, lowered quality of problem-solving strategies, and lowered expectations for future success (as cited in Dweck, 2015). In the literature of teacher professional identity (TPI), different variables are discussed, but the role of teachers’ ASs and their possible relationship with TPI seem to be rarely researched.

The present investigation aimed to explore the relationship between the professional identity of Iranian English as foreign language (EFL) teachers and their AS and compared experienced and novice teachers in shaping their professional identity and sought to find the answers of these research questions:

Q_1_: Is there any significant relationship between the professional identity of Iranian EFL teachers and their AS?

Q_2_: Is there any significant difference between experienced and novice teachers’ professional identity regarding their AS?

## Literature Review

### Teacher Identity

Identity as a general term defined by Alsup (2006) is considered as a sense of selfhood and is the subject of change through the passage of time based on the context in which the individual lives and works. Core identity can provide “the ability to initiate action and to register experience” (Gilligan, 2003, p. 167). In line with the researchers’ definitions of identity, Alsup (2006) puts its development as a non-stopping, ever-changing phenomenon that mandates every individual to experience “discursive tension and cognitive dissonance leading to heightened understanding (meta-awareness) of the intersections among personal and professional subjectivities” (p. 205).

In the field of EFL/english as a foreign language (ESL) education, the subject of teacher identity is considered as a crucial factor affecting their performance and consequently receive and attract the attention of numerous prominent researchers (Stryker and Burke, 2000; Stout, 2001; Walkington, 2005; Alsup, 2006; Rorrison, 2008; Korthagen, 2013). Moreover, various outlooks have been considered by researchers, such as Gilligan (2003), Taylor (1989), Noddings (2005), Isaacs (2007), and Miller (2009), and. They defined teacher identity as a unique sense of self-carrying by an in-service teacher as a teacher that covers his/her broad attitudes toward teaching and learning.

Bullough (2011) asserts that decision-making of teachers in schools and in particular in the classroom is closely related to and affected by understanding the teacher identity, which forms the basis for his understanding of the profession. According to Alsup (2006, p. 14), teachers’ professional identity.

“Incorporates the cognitive, the emotional, the bodily, and the creative, such that to not allow (pre-service) student to talk about such issues, to not teach them how and why such issues are important to their teaching lives, to not give them the opportunity to speak and take the time to hear them are doing pre-service teachers and in-service teachers a disservice—we are leaving out, we are forgetting or choosing to forget, an important (if not the most important) part of being a teacher: the teacher identity.”

Even so, the development of teacher identity has been seen, at best, as a by-product of teacher education programs rather than as a targeted outcome (Alsup, 2006; Rorrison, 2008), and this is certainly the case from the perspective of pre-service teachers (Franzak, 2002).

Although it is a dominant belief that roles are externally shaped by others’ expectations, Colbeck (2008) asserted that “individuals define their own identities internally as they accept or reject social role expectations as part of who they are” (p. 10). Additionally, it is generally an accepted belief that identity is created, preserved, and modified through discourse and language (Gee, 2000; Varghese et al., 2005; Alsup, 2006).

In conclusion, personal and social identities are simultaneously considered as both integrated and separated issues. Olsen (2008) illuminated that by separation, the researchers mean that identities ceaselessly affect each other. In other words, identity can be considered as a product, related to the particular conditions, and a constant flow that affected uninterruptedly by external and internal factors. Sugrue (2005) discussed that identity is not individual and permanent. Individuals may preserve their behaviors and routines but assuredly are significantly influenced by outside factors.

### Teachers’ Professional Identity

In recent years, several scholars have found that TPI is related to images of self (Motallebzadeh and Kazemi, 2018; Derakhshan et al., 2020a,b; Kalali Sani et al., 2021). In teaching, one’s conception of himself/herself as a person is interwoven with how he/she acts like a professional. The person cannot be separated from the profession; “*it seems unlikely that the core of the personal will not impact the core of the professional*” (Loughran, 2007, p. 112).

Defining identity in the educational milieu is so sophisticated that needs to make a balance between personal (self-image) and social (role at the real act of teaching) by any teacher (Volkmann and Anderson, 1998; Coldron and Smith, 1999). To highlight the undeniable importance of having more research in the realm of teacher identity, it is claimed that one of the best ways teachers can understand and clarify their professional, cultural, and political identities, and more significantly, their language teaching and learning is to understand their identity (Varghese et al., 2005).

Teacher professional identity in the Iranian context has been extensively researched regarding germane variables by means of different designs (Abednia, 2012; Hesamoddini, 2013; Zare-ee and Ghasedi, 2014; Kao and Lin, 2015; Abtahi and Motallebzadeh, 2016; Ghanizadeh and Ostad, 2016; Mirzaee and Aliakbari, 2017). The role of professional identity is studied in relation to personal and social factors, in general, and TPI in particular (Kalali Sani et al., 2021). Having positive and negative attributions to the events can influence the construction of teacher identity type, which is affected by positive emotions and characteristics and negative ones without any specific borderline for segregation of positivity and negativity (MacIntyre and Gregersen, 2012).

### Attributional Styles

The most agreed definition of AS is provided by Reiland (2020), who believes that AS is the consistent way by which people can explain the reasons for the occurrence of good or bad events. Forty years ago, Hitro and Seligman (1975) proposed the “learnt helplessness model of depression, which proposed that control over the environment is a fundamental need for any organism, and if one is repeatedly exposed to unavoidable painful stimuli, one will come to expect that such events are uncontrollable and develop hopelessness and depression as a result” (P. 125). This model was later reformulated to the AS theory (Abramson et al., 1978), which identified three dimensions of attribution of positive and negative outcomes: (a) internality; (b) stability; and (c) globalism. The internality refers to the internal/external reasons for the occurrence of events, regardless of their positivity or negativity. Stability refers to the existence of the causes in the future and globalism means the existence of the causes for the event in a specific situation or not.

Seligman (1993, as cited in Peterson and Park, 2007) argues that repeated exposure to governable events might foster associate degree optimistic informative style, whereas continual exposure to uncontrollable events might foster a negative AS. Trust in social relationships is argued to create an associate degree optimistic informative style. Attribution theory is concerned with how and why ordinary people explain events and situations as they do. Correspondent inference theory was developed by Jones and Davis (1965), which accounts for a person’s inferences regarding an individual’s bound behavior or action. The most important purpose of this theory is to undertake and make a case for why individuals create internal or external attributions. Internal attribution is easily apprehensible because of the correspondence we see between motive and behavior.

The recent investigations of positive psychology in second language acquisition (SLA) (Seligman and Csikszentmihalyi, 2014) have roots in the tenets of positive personality characteristics and positive experiences and positive institutions. Gabry’s-Barker (2021) classified the research in the realm of positive emotions in the following levels: (a) individuals, such as positive personality characteristics or traits, (b) the subjective, such as positive emotional experiences, and (c) the group, such as positive support provided by institutions and society. ASs have been categorized as considering events based on positive and negative views of individuals. Positive point of view of teachers toward events can influence their emotions and character (MacIntyre, 2016) and consequently, these positive feelings and personality factors may lead to empathy, enjoyment, contentment, optimism, tolerance, love, satisfaction, self-efficacy, and success (Seligman, 2011; Fathi et al., 2021; Wang et al., 2021).

### Teaching Experience

Brandenburg et al. (2016) assert that it is publically believed there is a strong relationship between teachers’ years of teaching experience and their quality of teaching in a different context. They found no evidence of lower teaching quality for novice teachers (up to 3 years of teaching experience), but there were symptoms of low teaching quality for teachers with 4–5 years of experience; however, research has proved that regarding different factors this relationship may vary (Klassen and Chiu, 2010). Inconsistent conceptions used to describe categories of experience are some of the problems in the realm of teaching experience. For instance, the terms “graduate,” “beginning,” and “early career” are used confusingly interchangeable to describe teachers with experience of up to 5 years (Mockler, 2018; Sullivan et al., 2019). What makes this confusion stronger is the terms like “beginner/experienced” and “novice/expert” (Palmer et al., 2005). In the present study, the researchers classified experienced teachers as those with at least 5 years of teaching experience and novice teachers as those with fewer than five.

## Materials and Methods

### Participants and Setting

To calculate the population of Iranian EFL teachers for selecting the participants, the researchers estimated the total number of EFL teachers from the head offices of more than 10 provinces as 13,000. The proper number of participants for valid generalization of the results was calculated as 380 teachers teaching in different cities of all provinces of Iran, such as Khorasan Razavi, Tehran, Fars, East Azerbaijan, Lorestan, and Boushehr, teaching in junior and senior high schools. They were in different age range (22–47), selected regardless of their gender with academic degrees from AD to Ph.D., and they taught English at different levels with different proficiency and performance. They spoke Turkish as their native language in West and East Azerbaijan and Ardebil and the rest spoke Farsi as their first language.

### Instruments

#### Attributional Style Questionnaire

A highly demanding questionnaire in the realm of personality traits has been proposed, developed, and validated by a team of researchers (Peterson et al., 1982) to measure the general AS index. The researchers of the present study measured Iranian EFL teachers’ general ASs, which is a self-report instrument and indicates scores for generally 12 statements, i.e., six bad (negative) and six good (positive) events. This questionnaire consists of three causal dimensions that includes two dichotomies: internal vs. external, stable vs. transient, and global vs. specific causes. ASQ used a 7-point continuum for each of the three causal dimensions. The total number of ASQ items was 60, which was first developed and validated by Peterson et al. (1982). They measured the ASQ construct validity and reported a satisfactory index. To measure the reliability index of ASQ, the test-retest method was used and it proved that total scores over a 5-week period were adequate (0.64 for negative events and 0.70 for positive events; Peterson et al., 1982). The results of Cronbach’s alpha analysis for measuring the reliability index of ASQ were calculated as 0.73. Table 1 shows the Cronbach’s alpha indices for TPI and AS, which were 0.89 and 0.80, respectively, and are satisfactory indices for the questionnaires.

**TABLE 1:** Reliability of the TPI and AS questionnaires.

Questionnaire	Cronbach’s Alpha	N of Items
AS	0.80	36
TPI	0.89	19

*TPI, teacher professional identity; AS, attributional style.*

#### Teachers’ Professional Identity Questionnaire

To measure Iranian EFL teachers’ index of professional identity, the researchers employed a TPI questionnaire, which has been first developed and validated by Cheung (2008). It first consisted of 41 items, but after its modification and measuring the validity through Exploratory and Confirmatory Factor Analysis (EFA, CFA), Cheung kept 19 items using a 5-point Likert-point scale ranging from “very weakly” to “very strongly” with a satisfactory Cronbach’s alpha index (α = 0.83). The readability and validity of teacher professional identity scale (TPIS) were double checked by three experts in applied linguistics. The total estimated time required to answer the items was about 20 min to read them carefully and choose their desired response.

### Procedure

Along with many problems of the pandemic of the Coronavirus Disease-2019 (COVID-19) virus all over the world, there was a big limitation for collecting the data by attending, visiting, and having face-to-face interaction for most of the research in education. To avoid the danger of infection, the TPI and AS questionnaires were converted to Google forms, and the links of the questionnaires were sent to EFL teachers in different provinces of Iran. At the beginning of the questionnaires, the explanations of the research aim and scope accompanied with the demographic information items of the respondents, such as their age, gender, academic degree, and years of teaching experience, were presented. The *ASQ* (Peterson et al., 1982) was sent to the participants. Moreover, their professional identity was measured through Cheung’s TPIS (Cheung, 2008). Collected data were classified based on the teachers who responded to the question in order to have accurate demographic information and their relevant answers to the items on questionnaires, the responses were transformed to specific values in the Statistical Package for the Social Sciences (SPSS) to calculate the probable relationships between teachers’ ASs with their professional identity. Moreover, the difference between teachers’ professional identity regarding experienced and novice teachers was measured by running an independent samples *t*-test.

### Design and Data Analysis

Two major variables of the present study were TPI and their AS, thus the design of the present study was a correlational design to measure their relationship. Moreover, this research sought to investigate the differences between the professional identity of Iranian EFL novice and experienced teachers. Since the purpose was to find the difference, independent samples tests were also used.

## Results

Before analyzing the collected data to answer the research questions, the researchers measured the reliability indices of both questionnaires by running Cronbach’s alpha. Moreover, a Kolmogorov-Smirnov test was performed to measure the normality of data distribution. In order to answer the first research question, a Spearman correlation coefficient (based on the normality) was calculated. The main concern of the second research question was to compare TPI of novice and experienced teachers, thus, an independent samples *t*-test was run. The following presents the tables and descriptions for data analysis.

One of the problems each researcher may face with every set of data is to have unengaged participants who carelessly answered the items with marking only one option for all items. To clean the data, the answers provided by these participants should be removed. After cleaning the data collected from 317 participants who returned the questionnaires, the answers of 40 participants were removed and 277 teachers were kept in the study. It is worth mentioning that one of the reasons why this happened to AS questionnaire could be the number of items (36). Table 2 shows that the K-S index (0.000) is significant for AS, thus the data violate the assumption of the normality. The index of “df” shows the number of participants (277) who answered the items on AS questionnaire. The relationship between TPI and AS should be calculated by running a Spearman test since the data set is not normal.

**TABLE 2:** Normality test for AS and TPI.

	Kolmogorov-Smirnov		
	Statistics	Df	K-S
AS	0.156	277	0.000
TPI	0.657	277	0.200

*TPI, teacher professional identity; AS, attributional style.*

As mentioned above, since the data were not normal, the researcher analyzed the data non-parametrically. Table 3 shows that there is a direct (0.12) and significant (0.04) relationship between AS and TPI. Thus, it can be concluded that if teachers have a higher index of ASs, they will also have a higher index of TPI.

**TABLE 3:** Spearman rho Correlation for AS and TPI.

		TPI	AS
Spearman’s rho	TPI	1.000	0.12
			0.04
		277	277

*TPI, teachers professional identity; AS, attributional style.*

The second research question deals with the possible significant difference between novice and experienced teachers regarding their professional identity. To answer this question, teachers were grouped based on their years of teaching experience. The first group was composed of those teachers with more than 5 years of teaching experience (labeled as experienced), and the second group was composed of those teachers with less than 5 years of teaching experience (labeled as a novice). In order to compare teachers’ professional identity based on their teaching experience, an independent sample *t*-test was run. The results of the germane analyses were shown in the following tables:

Table 4 shows the number of experienced and novice (*N* = 178, 99) teachers’ data of their professional identity. Moreover, their mean scores on the TPI questionnaire (*M* = 78.30, 62.34), their *SD*s = 8.04, 11.76 were shown.

**TABLE 4:** Descriptive statistics for experienced and novice teachers’ TPI.

	Experience	N	Mean	SD
TPI	Experienced	178	78.30	8.04
	Novice	99	62.34	11.76

*TPI, teachers professional identity; AS, attributional style.*

The prerequisite for considering the output of data in the independent sample *t*-test is to check the value of *p*. The results of the *F*-test determined whether to use the equal variances assumed rows or the equal variances not assumed rows in evaluating the t statistic. Since the significance level (Sig. = 0.000) for the *F*-value is less than 0.05, thus, the variances of the two groups are not equal, and therefore, the output in the equal variances *not* assumed row should be used. The results of Table 5 indicate that there is a significant difference between the TPI of experienced and novice teachers, *t*(149) = 12.02, *p* = 0.000. That is, the average performance score of experienced teachers (*M* = 78.30, *SD* = 8.04) was significantly different from that of novice teachers (*M* = 62.34, *SD* = 11.76).

**TABLE 5:** Independent sample *t*-test for experienced and novice teachers’ TPI.

		Levene’s test for equality of variances		*t*-test for equality of means			
		*F*	Sig.	*t*	df	*p*-value	Mean difference
TPI	Equal variances assumed	36.56	0.000	13.35	275	0.000	15.95
	Equal variances not assumed			12.02	149.93	0.000	15.95

*TPI, teacher professional identity.*

## Discussion

The main purpose of this study was to explore the relationship between Iranian EFL teachers’ ASs and their professional identity. Moreover, the researchers aimed to compare the novice and experienced teachers’ professional identity. The results obtained from the correlational analysis revealed that there is a positive (0.12) and significant (0.04) relationship between teachers’ ASs and their professional identity. The history of TPI studies shows that it has been researched from various angles considering dimensions related to personal and educational factors, such as motivation and autonomy (Murray et al., 2011), goal orientation (Kalali Sani et al., 2021), self-esteem (Motallebzadeh and Kazemi, 2018), and critical thinking (Sheybani and Miri, 2019), but there was no trace of general ASs, were detected. It may be fruitful to know whether teachers attribute their (and their learners’) success and failure to internal or external causes, or these causes are stable or transient, or they are global or specific, and how much these causes can be significant in shaping the professional identity of teachers.

The results of this study are in line with a similar study in the Iranian context conducted by Ghonsooly et al. (2015) who investigated EFL teachers’ attributions of success and failure. Their results showed different aspects regarding English language teachers’ attributions of success and failure events. They also found that these attributions vary by their age, teaching experience, and educational level, but not by gender. In the case of external/internal attributions, which are similar to the concepts of Locus of Control (LOC), Toussi and Ghanizadeh (2012) conducted a study and found that the variation in teacher self-regulation can be explained by taking their internal LOC into account. The findings of the present study also emphasized the significance of internal attributions in shaping teachers’ professional identity.

Wang and Hall (2018) conducted a review study about teachers’ causal attributions, and Weiner’s (2010) attribution theory was studied in 79 other research projects. Their findings showed a significant relationship between teachers’ attributions and their emotions and cognitions. Moreover, they came up with the prevalence and implications of teachers’ causal attributions to be moderated by critical background variables. The findings of this study are similar to Wang and Hall’s study in the case of the significant relationship between teachers’ ASs and some cognitive factors, such as professional identity.

The other variable investigated in this study was years of teaching experience. Novice and experienced teachers were compared in terms of their TPI index and the results revealed that experienced teachers enjoyed a higher index of TPI. The impact of experience has been investigated in many studies about teachers, such as teaching quality (Araujo et al., 2016; Sullivan et al., 2019); teacher effectiveness (Karimvand, 2011; Kini and Podolsky, 2016); and teacher creativity (Cremin, 2009; Avila, 2015). The findings of the present study were in line with most studies in which the results revealed the significant impact of years of teaching experience on the aforementioned variables.

## Conclusion and Implication

The main concern of the first research question was to investigate the relationship between teachers’ ASs and their professional identity, and the results of the Spearman index revealed a positive and significant relationship between teachers’ ASs and their professional identity. Identity construction in a specific profession needs to create a self-image, which is personally constructed and socially co-constructed. This can be achieved through the early stages of teacher education and developed through teaching empowerment courses. The undeniable role of teachers in shaping the future of the society suggests that TPI construction should be assessed continuously, which helps teacher educators design materials in harmony with their needs in teacher education programs and update their demands based on the twenty-first century principles (Hanna et al., 2020). Developing positive, internal, stable, and global styles in EFL teachers may help them shape stronger TPI and prepare teachers for the ever-changing world with the new wants of the young generation. On the other hand, negative attributions should be carefully treated to construct a fine-tuned identity for learners with ASs, such as external, transient, and specific.

The second research question dealt with the impact of experience on Iranian EFL teachers’ professional identity. The results of the independent samples *t*-test were shown that experienced teachers enjoy a higher index of TPI. It can be concluded that teachers construct and reconstruct their TPI through the years of teaching experience. The mixed findings of studies about teaching experience suggest that pre-service teachers need education in the realm of the positive and negative psychological (attributional) factors for constructing and shaping their identity to have adequate readiness for the real classes. This can be operationalized in having classes with experienced teachers to help novice ones to understand challenging circumstances to decide what factors can be beneficial for learners to look upon. They can also give novice teachers insights about how they can analyze events in the real classes and overcome probable difficulties.

## Data Availability Statement

The raw data supporting the conclusions of this article will be made available by the authors, without undue reservation.

## Ethics Statement

Ethical review and approval was not required for the study on human participants in accordance with the local legislation and institutional requirements. The patients/participants provided their written informed consent to participate in this study.

## Author Contributions

All authors listed have made a substantial, direct, and intellectual contribution to the work, and approved it for publication.

## Conflict of Interest

The authors declare that the research was conducted in the absence of any commercial or financial relationships that could be construed as a potential conflict of interest.

## Publisher’s Note

All claims expressed in this article are solely those of the authors and do not necessarily represent those of their affiliated organizations, or those of the publisher, the editors and the reviewers. Any product that may be evaluated in this article, or claim that may be made by its manufacturer, is not guaranteed or endorsed by the publisher.
